# Impact of schizophrenia and schizophrenia treatment-related adverse events on quality of life: direct utility elicitation

**DOI:** 10.1186/1477-7525-6-105

**Published:** 2008-11-28

**Authors:** Andrew Briggs, Diane Wild, Michael Lees, Matthew Reaney, Serdar Dursun, David Parry, Jayanti Mukherjee

**Affiliations:** 1 Oxford Outcomes Ltd, Oxford, UK; 2 Section of Public Health and Health Policy, University of Glasgow, Glasgow, UK; 3 Neuroscience and Psychiatry Unit, The University of Manchester, Manchester, UK; 4 Bristol-Myers Squibb Company, Uxbridge, UK

## Abstract

**Objective:**

To examine the impact of schizophrenia, its treatment and treatment-related adverse events related to antipsychotics, on quality of life from the perspective of schizophrenia patients and laypersons.

**Methods:**

Health state descriptions for stable schizophrenia, extra pyramidal symptoms (EPS), hyperprolactinemia, diabetes, weight gain and relapse were developed based on a review of the literature and expert opinion. The quality of life impact of each health state was elicited using a time trade-off instrument administered by interview to 49 stable schizophrenia patients and 75 laypersons. Regression techniques were employed to examine the importance of subject characteristics on health-related utility scores.

**Results:**

Patients and laypersons completed the interview in similar times. Stable schizophrenia had the highest mean utility (0.87 and 0.92 for laypersons and patients respectively), while relapse (0.48 and 0.60) had the lowest mean utility. Of the treatment-related adverse events, EPS had the lowest mean utility (0.57 and 0.72, respectively). Age, gender and PANSS score did not influence the utility results independently of health state. On average, patient utilities are 0.077 points higher than utilities derived from laypersons, although the ranking was similar between the two groups.

**Conclusion:**

Events associated with schizophrenia and treatment of schizophrenia can bring about a significant detriment in patient quality of life, with relapse having the largest negative impact. Results indicate that patients with stable schizophrenia are less willing to trade years of life to avoid schizophrenia-related symptoms compared to laypersons. Both sets of respondents showed equal ability to complete the questionnaire.

## Background

Schizophrenia is a severe form of mental illness affecting approximately 24 million people worldwide [1]. Clinical management of schizophrenia is possible using a range of different antipsychotics, although treatment is associated with a variety of treatment-related adverse events. In order to capture the true impact of treatment benefit, it is important to quantify not only the impact of the disease on health-related quality of life, but also the impact of treatment-related adverse events.

Previous studies investigating the impact of schizophrenia on quality of life have focused on the different stages of the disease and extra pyramidal symptoms (EPS) such as akathesia, agitation, and tardive dyskinesia [2,3]. However, with the introduction of an increasing number of atypical antipsychotics (such as aripiprazole, olanzapine, risperidone and quetiapine), differences between treatments related to adverse events, such as hyperprolactinemia and weight gain, are also important. One naturalistic study shows clear differences in the incidence of these adverse events between different atypicals, as well as differences between typical and atypical antipsychotics [4]. Other research has shown that after adjustment for personal risk factors and concomitant drug-use, patients taking conventional or newer anti-psychotics have a significantly increased risk of diabetes [5]. Such differences are potentially important drivers of the relative cost-effectiveness of different antipsychotics for the treatment of schizophrenia.

With the exception of EPS, the impact of these adverse events and of treatment relapse on quality of life has not been previously assessed. For health economic evaluation health related utilities are the preferred method of measuring health related quality of life with values ranging from less than zero to 1, where zero represents death and 1 represents best possible health. The most commonly used approach to eliciting utilities is the time trade-off technique (TTO) [6] and this approach has also been applied in schizophrenia [7]. When eliciting utilities for use in health economic evaluation, the choice of whether to elicit values direct from patients or from the lay population is contentious [8]. On the one hand, patients have direct experience of the disease and should therefore be able to give a more informed response as to the burden of health states that they have actually experienced. Laypersons, on the other hand, have no vested interests in one particular disease and can be thought of as 'future patients'. The National Institute for Health and Clinical Excellence (NICE) in the UK, clearly recommends that the utilities be elicited based on public preferences in its guidance [9].

In order to be consistent with the NICE reference case, but also to reflect the lack of agreement in the literature, in this study we chose to elicit utilities directly from both lay and patient groups using the time trade-off approach. By doing so it was possible to assess how these two groups compared in terms of ability to complete the exercise as well as the utilities obtained for the disease itself and the treatment related side-effects. In addition, we also administered the EQ-5D instrument to patients (EuroQol Group, 1990) [10]. The EQ-5D is a descriptive instrument that describes the health status of a patient and can be used to obtain an indirect estimate of utility by employing utility tariffs derived from a large scale lay population sample using the TTO method (Dolan et al, 1997) [11].

## Methods

### Descriptions of the health states

Health state descriptions for each of the schizophrenia-related symptoms and adverse events were developed to form the basis of the utility elicitation. These health states were developed and adapted according to the following approach:

1. Symptoms and potential adverse events were identified from a comprehensive review of the literature using Medline, and Embase databases, by considering comments on patient websites and in close consultation with Dr. George Awad, Professor of Psychiatry, University of Toronto. Health state descriptions were then developed based on these symptoms and adverse events,

2. Cognitive interviews were conducted with ten patients with schizophrenia. Cognitive interviews (Willis 1999) [12] are designed to assess comprehension and the cognitive processes undertaken by the respondent to answer a question. There are two major sub-types of cognitive interviewing methods: Think-aloud and verbal probing. This study utilised a verbal probing approach to assess the comprehensibility, clarity and relevance of the health states to patients. Feedback was provided by patients on the wording of the health states.

3. A cognitive debriefing study was also conducted with ten laypersons to assess the comprehensibility and clarity of the health state descriptions. Feedback was provided by respondents on the wording of the health states.

4. Finalisation of the health states was based on the feedback provided by patients and lay respondents.

This approach ensured that the health state descriptions were clinically relevant and meaningful and that health-related utilities elicited from patients and laypersons could be compared. Table 1 presents the final health state descriptions. Although the quality of life impact of EPS and relapse has been previously assessed [13-19] these health states were included so that the comparison between utilities for these and other health states was based on the same sample.

**Table 1:** Health State Descriptions for the TTO

Health State	Description
Stable schizophrenia – No side-effects	Base-Case Stable Condition^a^
	• I am in my mid-30's, living alone with no dependants.
	• My condition puts some limits on my daily life including the necessity to take regular medication. I have no problems with self-care and am able to complete household chores, but I don't meet too many new people.
	• I am able to work at a part-time paid or voluntary job.
	• Sometimes I hear things that no one else hears. I think someone is calling my name but when I turn around, no one is there. The things they say aren't scary, like when I was really sick, they are just calling my name.
	• Sometimes it feels like there are other people in my house that shouldn't be there, or that people go through my things without asking. I don't think about this most of the time though.
	No consequences from the treatment
Weight gain side-effect	Base-Case Stable Condition plus
	Consequences from the treatment:
	• In the last six months I have gained more than a stone in weight and it makes me pretty depressed as I find it very hard to lose weight by diet and exercise.
	• The extra weight has restricted my mobility and breathing and made some of my clothes too tight.
	• I am worried about my weight gain because I have heard that that this might cause diabetes, heart problems and make me lose a year or two off my life expectancy.
Diabetes side-effect	Base-Case Stable Condition plus
	Consequences from the treatment:
	• Since taking treatment I have been diagnosed with diabetes – my doctor says that it may be due to my treatment.
	• I have started to feel tired and need to urinate more often. I also seem to feel nauseous and get sick more often.
	• My doctor told me to change my eating habits so I have a more balanced diet, as well as drinking a maximum of two alcoholic drinks per day and taking my oral medication – otherwise the diabetes could get worse.
	• The doctors are also telling me to exercise a lot more than before. I need to always make sure that I have my medicine and something sweet with me in case I get dizzy or faint.
	• I need to test my blood sugar levels every day by pricking my finger with a pin and putting the blood on a paper strip.
	• My doctor told me about research showing people with diabetes might lose more than five years off life expectancy
Hyperprolactinemia side-effect (Male)	Base-Case Stable Condition plus
	Consequences from the treatment
	• I feel less interested in sex and when I do have sex, it is not as good as it was before I started treatment
	• My doctor tells me that there is also a good chance that my breasts will be bigger than other men's and that a little milk might sometimes come from them.
Hyperprolactinemia side-effect (Female)	Base-Case Stable Condition plus
	Consequences from the treatment
	• I feel less interested in sex and when I do have sex, it is not as good as it was before I started treatment
	• My doctor tells me that taking the treatment may make my periods not come when I think they will. The doctor also tells me that I might also have a little milk flow from my nipple when it shouldn't.
EPS side-effect	Base-Case Stable Condition plus
	Consequences from the treatment
	• Since I started treatment it seems as though I don't have full control over my muscles.
	• Often I feel that my muscles are quivering and I just can't seem to sit still, while other times it actually feels at though my muscles are undergoing spasms.
	• Other times it seems as though my body is moving when I don't want it to, and I do things like thrusting my tongue, marching up and down on the spot and humming.
	• Sometimes people say that I shuffle rather than walk, and that my face doesn't show any emotion.
Relapse	• My condition has forced me to go back to hospital for treatment, and not many people come to see me – not that I want to see anyone
	• It depresses me that I seem to have gone back to where I started before treatment, it seems as though there was no point in taking all those medicines
	• I am not able to work at the moment and I am worried that my employer will not want me back
	• I hear people calling me names and telling me to do things, just like I did when I was really sick
	• It feels like the other people in the hospital are watching me, and talking about me behind my back.

^a ^Base-Case Stable condition refers to a Typical patient with stable schizophrenia

### Participant recruitment

Both laypersons (n = 75) and patients (n = 50) were recruited to the study. Laypersons were recruited through newspaper advertising in Yorkshire, Oxfordshire and London in May and June 2005 and represented a convenience sample. Fifty adult outpatients with a diagnosis of schizophrenia or schizoaffective disorder (according to DSM-IV guidelines) who were experiencing stable symptoms were recruited to the study from Cromwell Community Mental Health Centre, Manchester.

The choice of an outpatient 'stable schizophrenia' population was selected based on previous studies in schizophrenia, and ensured that participation in the study did not compromise the patients' well-being [7,17]. The stability of potential participants was judged by the supervising clinician (and confirmed by a total Positive and Negative Syndrome Scale (PANSS) [20] score ≤ 70 during the interview) on the day of the interview. Ethics approval for patient involvement was gained from the Bolton Local Research Ethics Committee in February 2005, and the study was conducted in one-to-one interviews. Standard ethically approved procedures were applied to obtain consent where patients were asked to read the patient information sheet and were asked to sign the consent form if they were happy to participate in the study.

### Utility interview

#### Laypersons

After completing a demographic form, the 75 laypersons (a) read a short passage explaining schizophrenia and (b) viewed a DVD that explained the impact of schizophrenia on a person's life. The DVD showed an interview between a psychologist and a stable schizophrenic patient [21]. The patient is able to articulately recall the effect schizophrenia had on her life during acute exacerbations, and the symptoms that she still experiences.

A trained interviewer then asked subjects to complete a rating scale (visual analogue scale) where each health state is ranked along the preference assessment rating scale between scores of 100 (best possible health state) and 0 (worst possible health state). The rating scale was administered as an introductory task to familiarise respondents with the health states, as suggested by Torrance et al. [22], rather than to compare against utilities derived from the TTO. After completing the rating scale participants completed the TTO for each health state. Health states were presented in random order to ensure that results were not influenced by the order of presentation.

Each health state description was presented to the respondent. The interviewer offered a choice between spending 30 years in that state followed by death (Alternative 1), or 29 years with perfect health followed by death (Alternative 2). A 30-year time frame reflects the average life expectancy of schizophrenia patients in the hypothetical health state descriptions. If subjects chose to spend 30 years in the health state being valued, they were then asked to choose between 30 years in that health state (Alternative 1), followed by death, or 1 year in perfect health followed by death (Alternative 2). This is known as the 'ping-pong method' [23] and is a standard approach to health-related utility elicitation. The process continued until the respondent was indifferent between spending 30 years in the particular health state and the number of years being offered in perfect health. This process was repeated for each health state being valued.

#### Patients

The 50 patients self-completed a demographic form and the EQ-5D utility questionnaire to assess the health-related utility of the patient for the day of the interview [10]. The EQ-5D questionnaire produces answers to five questions. This combination of answers, which provides the patient mapped functionality of their current condition, then maps to a utility score that has been generated from a UK lay population [11]. The result of this questionnaire provides an important validation of the baseline health state – stable schizophrenia – described in this study.

Clinical data (relating to the patients' medical history) were also collected and a trained mental health nurse administered the PANSS interview to assess the level of the patients' symptomatology. One patient with a PANSS score of 83 was excluded from further analysis. A trained interviewer then administered the same interview to the 49 remaining patients to elicit TTO utilities for the different health states exactly as was described above for the lay sample.

### Statistical methods

Mean utility scores from the TTO method [22] and standard deviations/errors were calculated for each health state. In addition, a random effects regression analysis was performed where random effects controlled for repeated measures on the same subjects valuing different health states. The regression was used to determine whether patients and laypeople report different utility values, and to determine whether utility values are explained by the demographics of the lay sample or the PANSS score for the patient sample. Potential explanatory variables that were not significant predictors of utility score were omitted from the regression analysis on the grounds of parsimony, but only after applying a test of joint significance of excluded variables. Robust standard errors were reported in order to account for heteroskedasticity. All regression analyses were performed using STATA 8.0.

## Results

The study sample consisted of 75 laypersons and 49 patients which makes it the largest utility study to date in this disease area. All patients and laypersons completed the utility interview. Demographic characteristics are presented in Table 2. As would be expected, people with schizophrenia appear to have problems holding down employment: 62% percent of the lay sample was in full-time or part-time paid work opposed to only 8% of the patient population. Patients were also more likely to be single and have achieved a lower educational attainment. Seventy percent of patients were diagnosed with paranoid schizophrenia and a further 27% diagnosed with schizoaffective disorder. Olanzapine, quetiapine and clozapine were the most commonly prescribed antipsychotic medications, although patients were often treated using more than one antipsychotic. Total PANSS scores range between 30 and 64. Patients were diagnosed between the ages of 15 and 51, with the mean age of diagnosis 25.9 years (SD = 7.59).

**Table 2:** Population Characteristics

Characteristic	Layperson Sample	Patient Sample
Total number (n)	75	49
Male/Female (n)	35/40	22/27
Mean age (years)	39.4 (17–76)	43.5 (21–64)
White ethnicity (%)	93.3%	93.9%
		
Marital Status		
Single	21.3%	51.0%
Married	65.3%	30.6%
Cohabiting	8.0%	12.2%
Divorced	2.7%	2.0%
Widowed	2.7%	4.1%
		
Highest educational level		
Did not complete high school	1.3%	28.6%
Minimum school age (GCSE's)	24.0%	59.2%
A-Levels	10.7%	8.2%
Degree or equivalent qualification	52.0%	4.1%
MSc Degree/PhD	12.0%	0%

The mean time taken to complete the utility interview was 26 minutes for both the lay and patient samples. Utilities derived using the time trade-off approach are presented for both patients and laypersons in Table 3. These show that laypersons and patients both view relapse and EPS as being the least desirable health states in which to spend time, followed by diabetes. There is little difference between the utilities associated with time spent with hyperprolactinemia or weight gain, and the ordering of these symptoms is reversed between patients and laypersons. The stable disease state was considered to have the highest utility by both groups.

**Table 3:** Time trade-off utilities for lay and patient samples

Health State	Mean utility (standard error)		T-test for difference*
		
	Patient sample	Lay sample	
Stable schizophrenia	0.919 (0.023)	0.865 (0.021)	p = 0.087

Weight gain	0.825 (0.028)	0.779 (0.024)	p = 0.216

Diabetes	0.769 (0.036)	0.712 (0.028)	p = 0.215

Hyperprolactinemia	0.815 (0.030)	0.783 (0.025)	p = 0.415

Relapse	0.604 (0.042)	0.479 (0.033)	p = 0.022

EPS	0.722 (0.037)	0.574 (0.032)	p = 0.003

*Unequal variance t-test

Table 3 also shows patients reporting significantly higher utilities (*p *< 0.05) for stable schizophrenia, EPS and relapse than laypersons, while there are near significant differences for weight gain and diabetes. However, a joint assessment of the overall differences between results from the patient and lay populations should take into account the repeated utility measurement from individual subjects. Table 4 therefore presents the results of two multiple regressions, which shows the impact of each health state, gender, age and respondent group (patients or laypeople) on the utility value. The constant term represents the utility associated with stable schizophrenia valued by a layperson and coefficients reported represent changes in utility relative to this value.

**Table 4:** Determinants of utility values

Explanatory variable	Coefficient (standard error) – Unrestricted regression	Coefficient (standard error) – Parsimonious regression
Constant	0.794 (0.062)*	0.856 (0.021)*

Weight gain	- 0.090 (0.021)*	- 0.089 (0.015)*

Diabetes	- 0.151 (0.021)*	- 0.151 (0.019)*

Hyperprolactinemia	- 0.087 (0.021)*	- 0.089 (0.014)*

Relapse	- 0.355 (0.021)*	- 0.358 (0.025)*

EPS	- 0.256 (0.021)*	- 0.256 (0.022)*

Patients	0.071 (0.034)*	0.077 (0.033)*

Age	0.002 (0.001)	na

Female	- 0.039 (0.033)	na

Diagnostic parameters (unrestricted regression):

Number of observations = 738.

R^2 ^(within) = 0.3871; R^2 ^(between) = 0.0726; R^2 ^(overall) = 0.2339.

Wald chi^2^(8) = 394.56.

Prob > chi^2 ^< 0.0001.

Joint significance test on "Age" and "Female": chi^2^(2) = 3.81; Prob > chi^2 ^= 0.1492

Diagnostic parameters (parsimonious regression):

Number of observations = 738.

R^2 ^(within) = 0.3891; R^2 ^(between) = 0.0424; R^2 ^(overall) = 0.2215.

Wald chi^2^(6) = 397.18.

Prob > chi^2 ^< 0.0001

It is clear from the first regression model that age and gender have no significant influence on the utility and these were therefore excluded from the model (having established they were not jointly significant). The resulting model shows that, on average, patient utilities are 0.077 points higher than utilities derived from laypeople. These results also show that time spent in the relapse and EPS states is associated with reductions in utility of 0.358 and 0.256 points, respectively.

An additional regression analysis was performed for the patient sample only, including PANSS score as a predictor. The results showed no evidence that the PANSS score had any influence on the utility score reported by patients, although the power of this test is reduced by the reduction in sample size related to restricting the regression to just 49 patients.

The mean utility associated with the patient population's current state of health, as measured by the EQ-5D, was 0.86. This health-related utility value is lower than the utility for stable schizophrenia elicited directly from the patient population (0.92), but almost identical to the utility for stable schizophrenia elicited from the lay population (0.865).

## Discussion

This study has demonstrated two important results. Firstly, that stable schizophrenia has the lowest impact on quality of life (highest utility value) and 'relapse' has the highest impact on quality of life (lowest utility value) of the health states measured. This is unsurprising, as these two states represent the extremes in schizophrenia-related health effects. The results also consistently showed 'EPS' to have the second-greatest impact on quality of life, followed by diabetes, while there was little difference between the quality of life impacts of 'weight gain', and 'hyperprolactinemia'.

The second key result is that the actual utility values varied considerably according to the population from which the values were derived. Utilities derived from patients were, on average, 0.077 points higher than those derived from the lay population. This indicates that patients are less willing to trade years of life to avoid schizophrenia-related health states. This is likely to be the result of a shift in psychological expectations, which includes a shift in the weight placed on different aspects of quality of life and a changed view of what matters in life. General population respondents are less likely to understand these shifts, tending to focus on the transition to the state rather than its longer term consequences, and therefore underestimate the ability of a patient experiencing the disease to adapt to their health state [8,24]. Other research indicates that general population respondents focus more on the negative aspects of a health state than the remaining positive aspects [25]. Together, these would lead to lower utility values from the general population than a patient population. These general observations, support schizophrenia-specific work that has pointed to the importance of self-experience and a model of recovery in this disease [26,27] which supports the general concept of adaptation.

The study results confirms the earlier work of Voruganti et al. [7] and Adams et al. [28] suggesting that stable patients are capable of participating in studies designed to elicit the quality of life impact of schizophrenia and its treatment. Despite differences in utility values, patients and laypersons took the same amount of time to complete the interview and interviewers reported no problems in understanding of the study tasks among either population. It is important in health services research to gain the perspectives of all participants, and this study shows that a well-designed, sensitively administered interview is able to elicit health-related utilities from patients as well as laypersons that can be used to inform decision makers about the quality of life impact of schizophrenia. It is likely that differences in the results reflect differences in perspective, rather than an inability of patients to provide appropriate responses.

The key potential problems in any health-related utility study (which limits the transferability of results) relate to the description of the health states and time period used as the benchmark in the time trade-off procedure. In the current study, the health state descriptions were developed after review of the published literature and consultation with clinical experts, and finalised following pilot studies with both patients and lay groups. Further, the mean utility values for stable disease – at 0.919 for patients and 0.865 for laypeople – were higher than hypothesised. However, the result from the EQ-5D patient scores was very similar to the utility for stable disease among laypeople. As noted previously, the utilities derived from EQ-5D scores are based on lay values. Hence, the similarity of the two results indicates that laypeople value the patient mapped functionality of their condition from EQ-5D at a very similar level to how laypeople value the stable schizophrenia health state described in Table 1. This provides a good indication that the health state descriptions are consistent with clinical reality and mitigates any concerns over the use of a convenience sample of laypersons in this study.

This study was designed to assess the impact on quality of life of key adverse events associated with the newer antipsychotics. Previous studies had shown that schizophrenia relapse has a substantial impact on quality of life, as does EPS. These results were supported in this study. However the adverse events primarily associated with the newer antipsychotics – hyperprolactinemia, weight gain and diabetes – have a lower impact on quality of life than EPS and relapse. There are two ways in which adverse events such as hyperprolactinemia, weight gain and diabetes – with the lower measured impact on quality of life – can affect the results of an economic evaluation. Firstly, such events are likely to influence the desire of patients and their families to continue with medication, and may cause patients to discontinue, with the associated increase in relapse. This would ensure that more time was spent in the relapse state with its substantial impact on quality of life. Secondly, the duration of these adverse events is also important. The impact of relapse on quality of life is substantial but relapse is a relatively transitory condition. Conversely, weight gain and diabetes show a smaller impact on quality of life than relapse, but are more sustained. The overall net effect of these quality of life differences could be determined through the use of the commonly employed outcome measure in health economic evaluations: the Quality Adjusted Life Year (QALY) which takes into account both the quality of life effects and the duration of that effect.

There are two main implications that flow from the results reported here. Firstly, that treatment-related adverse events all have a measurable impact on a patient's quality of life. While EPS and relapse have the greatest impact on quality of life, events such as hyperprolactinemia, weight gain and diabetes noticeably reduce patient quality of life compared with schizophrenia patients who do not suffer from these adverse events. These results offer the potential to minimise the net effect of disease and treatment on patient quality of life and quality-adjusted life years in economic analyses. Secondly, that the differences in valuations provided between patients and lay persons can be substantial in a disease such as schizophrenia and this could impact the cost-effectiveness of different treatment options for patients. Only by employing the sorts of estimates provided in this study in future cost-effectiveness models can the potential importance of these differences be fully determined.

## Conclusion

In conclusion, there were clear differences between patient and layperson responses to the utility questionnaire. However, the preference ordering of these health states was similar, with stable schizophrenia having the lowest impact on quality of life and relapse and EPS the greatest impact on quality of life, indicating as clear an understanding by patients of the health states and their impact on quality of life as by laypersons. In a disease such as schizophrenia, chronic side-effects of treatment such as weight gain and diabetes may have just as large an impact on QALYs as the acute symptoms of the condition itself.

## Competing interests

The authors declare that they have no competing interests.

## Authors' contributions

AB, DW, ML, DP, JM conceived and designed the study. SD oversaw data collection. ML and MR oversaw data collection and provided early drafts. AB oversaw the statistical analysis. DW, ML, MR and SD oversaw the development and testing of the vignettes. All authors provided critical comment through extensive drafting of the manuscript. DW is the guarantor for the study.
